# Dicer and microRNAs protect adult dopamine neurons

**DOI:** 10.1038/cddis.2017.214

**Published:** 2017-05-25

**Authors:** Piotr Chmielarz, Julia Konovalova, Syeda Sadia Najam, Heike Alter, Timo Petteri Piepponen, Holger Erfle, Kai C Sonntag, Günther Schütz, Ilya A Vinnikov, Andrii Domanskyi

**Affiliations:** 1 Institute of Biotechnology, University of Helsinki, Helsinki, Finland; 2 Institute of Pharmacology, Polish Academy of Sciences, Department of Brain Biochemistry, Krakow, Poland; 3 Laboratory of Molecular Neurobiology, Sheng Yushou Center of Cell Biology and Immunology, Department of Genetics and Developmental Biology, School of Life Sciences and Biotechnology, Shanghai Jiao Tong University, Shanghai, China; 4 Molecular Biology of the Cell I Division, German Cancer Research Center, Heidelberg, Germany; 5 Division of Pharmacology and Pharmacotherapy, University of Helsinki, Helsinki, Finland; 6 ViroQuant-CellNetworks RNAi Screening Facility, BioQuant, Heidelberg University, Heidelberg, Germany; 7 Department of Psychiatry, McLean Hospital, Harvard Medical School, 115 Mill Street, Belmont, MA, USA

## Abstract

MicroRNAs (miRs) are important post-transcriptional regulators of gene expression implicated in neuronal development, differentiation, aging and neurodegenerative diseases, including Parkinson’s disease (PD). Several miRs have been linked to PD-associated genes, apoptosis and stress response pathways, suggesting that deregulation of miRs may contribute to the development of the neurodegenerative phenotype. Here, we investigate the cell-autonomous role of miR processing RNAse Dicer in the functional maintenance of adult dopamine (DA) neurons. We demonstrate a reduction of Dicer in the ventral midbrain and altered miR expression profiles in laser-microdissected DA neurons of aged mice. Using a mouse line expressing tamoxifen-inducible CreERT2 recombinase under control of the DA transporter promoter, we show that a tissue-specific conditional ablation of Dicer in DA neurons of adult mice led to decreased levels of striatal DA and its metabolites without a reduction in neuronal body numbers in hemizygous mice (Dicer^HET^) and to progressive loss of DA neurons with severe locomotor deficits in nullizygous mice (Dicer^CKO^). Moreover, we show that pharmacological stimulation of miR biosynthesis promoted survival of cultured DA neurons and reduced their vulnerability to thapsigargin-induced endoplasmic reticulum stress. Our data demonstrate that Dicer is crucial for maintenance of adult DA neurons, whereas a stimulation of miR production can promote neuronal survival, which may have direct implications for PD treatment.

Dopamine (DA) neurons in the ventral midbrain project to striatal, limbic and cortical areas forming neuronal networks that regulate complex behaviors, emotions and controlled voluntary motion.^1^ A progressive age-related degeneration of mesostriatal DA neurons in the substantia nigra (SN) causes motor symptoms in Parkinson’s disease (PD).^2^ Selective vulnerability of DA neurons is attributed to number of factors, including mitochondrial dysfunction, endoplasmic reticulum (ER) stress, decreased neurotrophic factor signaling and increased oxidative stress.^3, 4, 5^ The exact cause of SN DA cell loss in sporadic PD is still unknown and the effectiveness of current treatment approaches is limited. As aging remains the strongest and most reliable risk factor in PD pathology,^6^ we conducted *in vivo* studies in aging mice to further understand the molecular mechanisms that contribute to the maintenance of adult DA neurons in the SN.

MicroRNAs (miRs) are approximately 22 nucleotides long non-coding RNA molecules that bind to complementary sites in target mRNAs and promote their degradation or suppress translation.^7^ MiRs can mediate a formation of large-scale regulatory networks and enable crosstalk between different cellular pathways.^8^ They have crucial roles in neuronal cell development and differentiation,^9^ and in the maintenance or age-related degeneration of neuronal cell systems, including DA neurons in PD.^10, 11, 12^ MiR expression changes were detected in brain samples from PD patients, suggesting that particular miRs can contribute to the development of the disease.^13, 14, 15, 16, 17^ As PD strongly correlates with age, affecting about 1% of people over 60 and 3% of people over 80 years,^6^ we hypothesized that age-related changes in miR expression may be a contributing factor to the selective vulnerability of DA neurons.

MiRs are processed from precursor molecules by two protein complexes containing double-stranded RNA-specific nucleases, Drosha and Dicer. Pri-miRs are initially cleaved in the nucleus by a complex containing Drosha and DiGeorge syndrome critical region gene 8 (DGCR8) proteins. In the cytoplasm, pre-miRs are further cleaved by Dicer in a complex with Argonaute 2 (Ago2), PACT and TAR RNA-binding protein 2 (TRBP) to produce mature miRs, which direct RNA-induced silencing complex to target mRNAs.^7, 18, 19^ During aging, Dicer is downregulated in the heart, adipose tissue and the brain.^20, 21^ The age-related decline in its expression, possibly caused by long-term cellular stress,^22, 23^ results in diminished miR processing and downregulation of mature miR levels.^20, 24^ Accordingly, miR expression profiling revealed a predominant decrease of miR levels in the aged mouse brain.^25^ Downregulation of mature miRs caused by dysfunction in miR biogenesis pathways has also been observed in motor neurons of amyotrophic lateral sclerosis (ALS) patients and in cells exposed to ER or oxidative stress.^18^ Interestingly, enoxacin – a small molecule interacting with TRBP and enhancing pre-miR cleavage in the Dicer-PACT-TRBP-Ago2 protein complex^19, 26^ – corrected ALS-related defects in miR biogenesis *in vitro,* and improved neuromuscular function in ALS mouse models.^18^

Disruption of the *Dicer1* gene in embryonic DA neurons severely impairs the development of the DA system.^14^ To specifically study miR functions in adult DA neurons, we generated a transgenic mouse model allowing conditional ablation of *Dicer1* using a tamoxifen (Tam)-inducible CreERT2 recombinase under control of the DA transporter (*Dat*, *Slc6a3*) promoter.^27, 28, 29, 30^ Compared with virus-mediated Cre expression approaches,^31^ the robust Tam-inducible CreERT2 system provides a high targeting efficiency and tight temporal and spatial control over recombination. In this study, we analyzed the cell-autonomous function of Dicer and the physiological and pathophysiological roles of the miR biogenesis pathway in adult DA neurons in the contexts of aging and PD. We show that Dicer-dependent miRs are crucial for the maintenance of adult DA neurons and that specific miRs are altered during aging. We further show that pharmacological stimulation of Dicer activity promoted survival of DA neurons in culture and attenuated their vulnerability to ER stress.

## Results

### Dicer is downregulated in the ventral midbrain of aged mice

**Figure 1:**
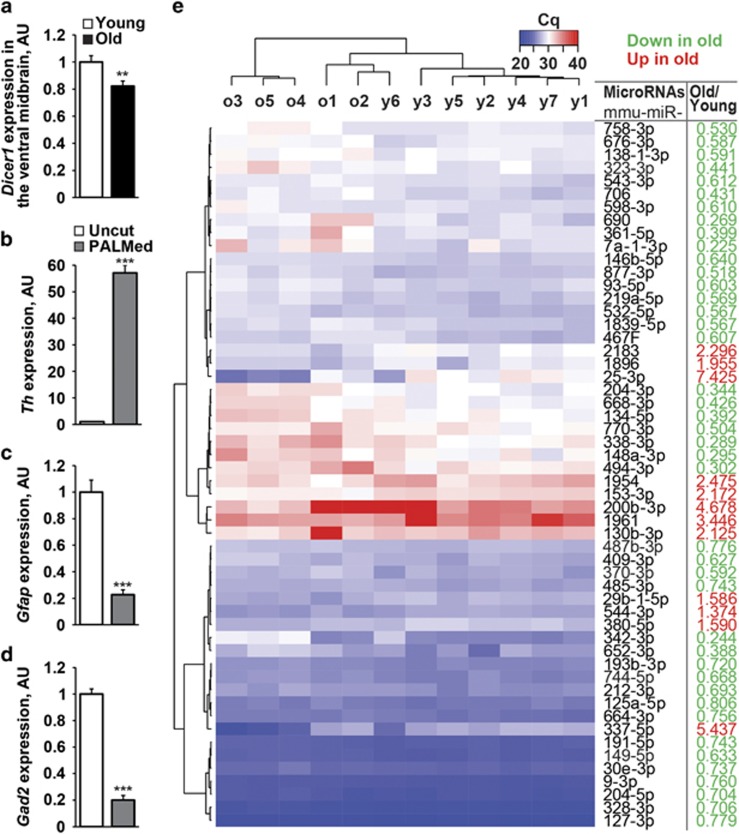
Changes of mRNA and miR expression levels in the ventral midbrain and laser-microdissected DA neurons from young and old wild-type mice. (**a**) Selective downregulation of *Dicer1* mRNA in the ventral midbrain of aged wild-type mice (*n*=6 and 8 for young and old animals, respectively). **P<0.01, as determined by Student’s unpaired two-tailed *t*-test. (**b-d**) Comparison of mRNA levels for *Th* (DA neuron marker), *Gfap* (astrocyte marker) and *Gad2* (GABAergic neuron marker) in RNA preparations from control ventral midbrain tissue (uncut) and laser-microdissected DA neurons (PALMed) (*n*=5 and 6, respectively). ***P<0.001, as determined by Student’s unpaired two-tailed *t*-test. (**e**) Heat maps showing expression levels, hierarchical clustering and fold change of 54 miRs with statistically significant changes (upregulated, red; downregulated, green; *P*<0.05, as determined by Mann–Whitney test) in microdissected DA neurons comparing old with young mice (*n*=5 and 7, respectively)

Aging is the strongest risk factor in sporadic neurodegenerative diseases, including PD. We assessed the expression of *Dicer1* and several other genes involved in the development and physiology of DA neurons by analyzing ventral midbrain samples from young (6.5 weeks) and old (87 weeks) wild-type C57Bl/6 N mice. The expression of typical DA neuronal genes and several members of neuron-associated signaling pathways did not significantly change with aging (Supplementary Figure S1A). In contrast, the levels of *Dicer1* mRNA were 20% downregulated in old animals (Figure 1a). Interestingly, in our previous studies on post-mortem laser-microdissected DA neurons, we observed a reduction of *DICER1* gene expression in PD patients (–1.9 fold, *P*<0.05) compared with age-matched healthy individuals, which was even more pronounced in the male population (–2.7 fold, *P*<0.05).^32^

### Predominant downregulation of miR expression levels in aged DA neurons

To explore if the observed downregulation of Dicer has led to changes in miR levels in aged DA neurons, we utilized an established protocol combining retrograde fluorescent-labeling with laser-assisted microdissection of DA neurons in young and aged mice.^4^ We first assessed sample quality by determining the expression of markers for DA neurons (tyrosine hydroxylase, *Th*), astrocytes (glial fibrillary acidic protein, *Gfap*) or GABAergic neurons (glutamic acid decarboxylase 2, *Gad2)* on RNA preparations isolated from the microdissected cells or surrounding ventral midbrain tissue. Compared with the latter, the relative amounts of *Gfap* and *Gad2* transcripts were depleted, whereas *Th* mRNA was markedly enriched in the microdissected DA neuronal samples (Figures 1b and d). These data confirm accurate microdissection and a high level of enrichment of DA neurons. We then used high-throughput TaqMan qPCR-based miR arrays that specifically detect the mature forms of miRs,^33^ to assess miR expression profiles in DA neurons from young and old C57Bl/6 N mice. This profiling identified 54 significantly changed miRs, most of which (42 miRs) being downregulated in the cells from old animals (Figure 1e and Supplementary Table S1). Notably, the expression levels of miR precursors remained unchanged in old compared with young mice (Supplementary Figure S1B), suggesting that the observed age-dependent decline of mature miRs is not caused by attenuated transcription of miR genes, but rather a consequence of impaired miR processing.

### Ablation of Dicer in adult DA neurons leads to their progressive degeneration and loss of striatal DA

**Figure 2:**
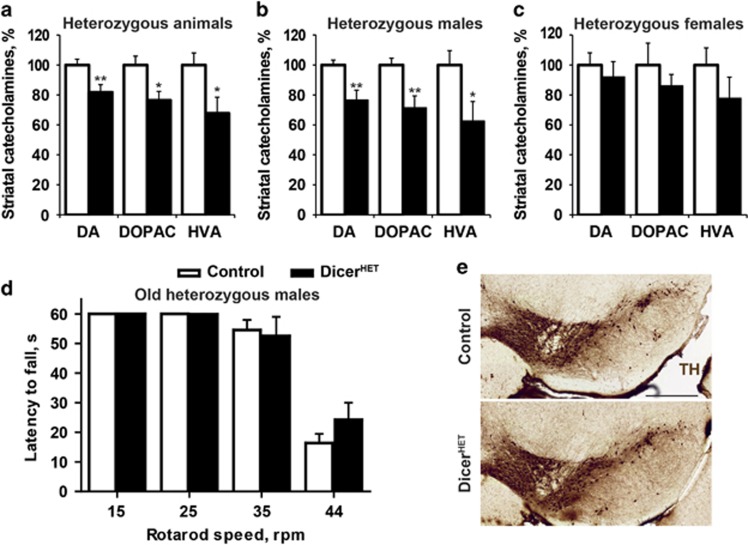
Inducible deletion of one *Dicer1* allele in adult DA neurons leads to decreased levels of striatal DA and its metabolites. (**a-c**) Quantification of striatal content of DA and its derivatives DOPAC and HVA in control and Dicer^HET^ mice (males: *n*=8 and 8, females: *n*=5 and 4, respectively) 11 weeks after start of Tam injections. (**d**) Mean latency to fall in a constant speed rotarod assay at 15, 25, 35 and 44 r.p.m. 90 weeks after start of Tam injections in control and Dicer^HET^ mice (*n*=4 and 4, respectively). (**e**) Immunostaining of the ventral midbrain sections from control and Dicer^HET^ mice 11 weeks after start of Tam injections. Scale bar, 500 *μ*m. *P<0.05; **P<0.01; in comparison with control mice, as determined by Student’s unpaired two-tailed *t*-test

To directly evaluate the importance of Dicer for the maintenance of adult DA neurons, we generated Dicer^fl/wt^/DATCreERT2 mice (henceforth referred to as *Dicer1* heterozygous conditional knockout, Dicer^HET^) and Dicer^fl/fl^/DATCreERT2 mice (*Dicer1* conditional knockout, Dicer^CKO^), in which the essential RNaseIII domain of the *Dicer1* gene is flanked with LoxP sites^30^ and the Tam-inducible CreERT2 recombinase is expressed in DA neurons under control of the *Dat* promoter.^27^ Induction of Cre recombination in 8- to 10-week-old Dicer^HET^ mice by Tam injections resulted in a sex-specific decrease of the levels of striatal DA and its metabolites 3,4-dihydroxyphenylacetic acid (DOPAC) and homovanillic acid (HVA) as early as 11 weeks after recombination (Figures 2a and c). Notably, similar to the asymptomatic stages of PD, mild decrease in DA levels upon gradual Dicer inactivation did not cause movement/posture disturbances (Figure 2d). Moreover, consistent with a male-directed bias in PD pathology,^17, 32^ the decreases in DA, DOPAC and HVA were significant in males, whereas reaching only tendency levels in females 11 weeks after Tam injections. Notably, at 19 weeks after recombination, we observed a statistically significant decline in the levels of striatal DA, but not DOPAC or HVA, in female Dicer^HET^ mice compared with controls (Supplementary Figure S1C), however, this decrease was not as pronounced as in male Dicer^HET^ mice at 11 weeks after Tam (Figure 2b). Importantly, Dicer^HET^ mice exhibited no loss of DA neurons in the ventral midbrain compared with age-matched controls (Figure 2e). These data show that, similar to an age-related *Dicer1* downregulation, its hemizygous inactivation causes functional deficits in DA neurons below the threshold when locomotor abnormalities become apparent.

**Figure 3:**
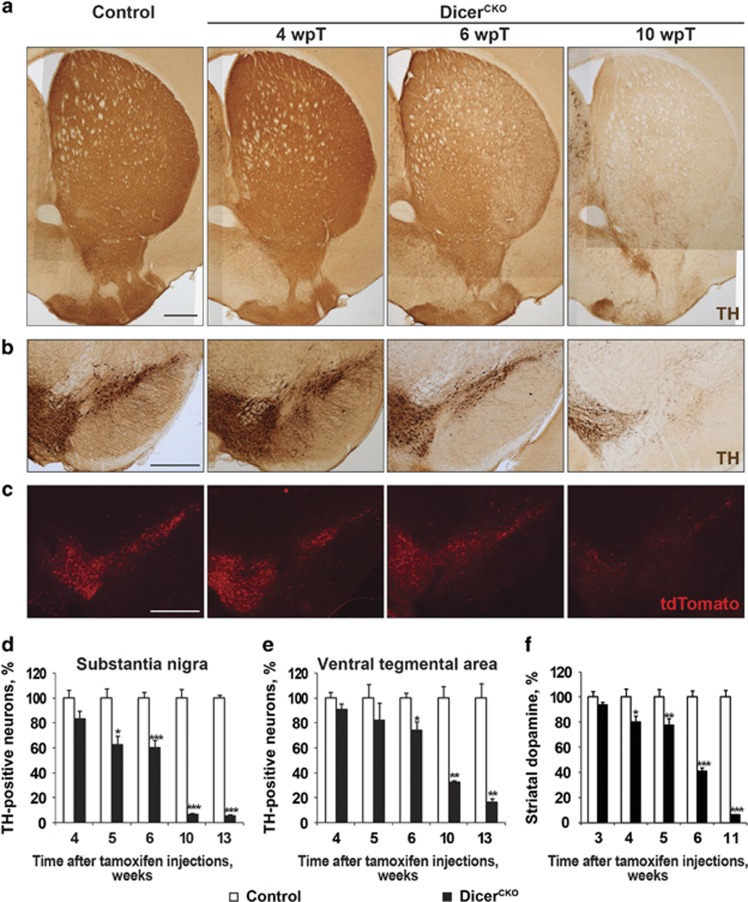
Progressive degeneration of adult DA neurons after inducible ablation of both *Dicer1* alleles. (**a-c**) Loss of the ventral midbrain DA neurons and their striatal projections at indicated times after the induction of *Dicer1* deletion by Tam injections (wpT, weeks post Tam) visualized by TH immunostaining or transgenic tdTomato fluorescence. Scale bar, 500 *μ*m. (**d** and **e**) Counts of TH-positive neurons in the SN (**d**) and ventral tegmental area (VTA) (**e**) in control and Dicer^CKO^ mice at indicated time points after start of Tam injections (for control and Dicer^CKO^ mice: *n*=7 and 9, 4 and 2, 8 and 6, 4 and 3, 4 and 3, respectively, for the indicated time points). (**f**) Quantification of striatal DA content for control and Dicer^CKO^ mice: *n*=5 and 4, 4 and 6, 6 and 8, 7 and 11, 12 and 18, respectively, for the indicated time points. *P<0.05; **P<0.01; ***P<0.001 in comparison with control mice, as determined by Student’s unpaired two-tailed *t*-test. wk, week

In contrast, inactivation of both *Dicer1* alleles resulted in a selective loss of its expression in adult DA neurons already 2 weeks after the start of Tam treatment (Supplementary Figure S1D). Dicer ablation led to a progressive loss of DA neurons in the ventral midbrain and degeneration of their axonal projections to the striatum (Figure 3). To confirm that the observed reduction in neuronal cell numbers was indeed caused by neurodegeneration and not by downregulation of TH, we monitored the fate of DA neurons in Dicer^fl/fl^/tdTomato/DATCreERT2 mice that express fluorescent reporter tdTomato upon Cre-mediated recombination selectively in DA neurons (Supplementary Figure S2),^34^ and immunostained the ventral midbrain sections with additional DA neuronal markers, such as Dat and DA decarboxylase (Ddc). Similar to our observations on TH immunoreactivity (Figures 3a, b and Supplementary Figure S3A), the number of neurons that were positive for Dat, Ddc and tdTomato was markedly reduced in Dicer^CKO^ animals (Figure 3c and Supplementary Figure S3B). Quantification revealed that Dicer^CKO^ mice lost about 90% of SN DA neurons 10 weeks after Tam injections, whereas the VTA region was more resistant to Dicer loss with some TH-positive neurons remaining up to 8 months after Tam treatment (Figures 3b, d, e and Supplementary Figure S3C). This pattern of cell death is also seen in clinical PD further corroborating a potential role of Dicer and miRs in PD pathogenesis. The selective degeneration of DA neurons was accompanied by a progressive loss of the striatal DA content (Figure 3f).

In accordance with the dynamics of DA neuronal loss in the SN and the VTA, Dicer^CKO^ mice exhibited elevated astrogliosis, as detected by increased anti-GFAP immunostaining at 6 and 10 weeks after Tam treatment (Supplementary Figure S4A-C). Increased oxidative stress is considered to be one of the main factors involved in selective degeneration of DA neurons in aging and PD.^3^ Consistently, after dihydroethidium injection, we detected an increase in ethidium fluorescence in TH-positive neurons of Dicer^CKO^ mice 4 weeks after Tam treatment (Supplementary Figure S4D), indicative of elevated mitochondrial ROS production.^35^ These data show that similar to other animal models^28^ and PD patients,^2^ increased oxidative stress also contributed to the degeneration of DA neurons in Dicer^CKO^ mice.

### Dicer^CKO^ mice exhibit progressive locomotor abnormalities

**Figure 4:**
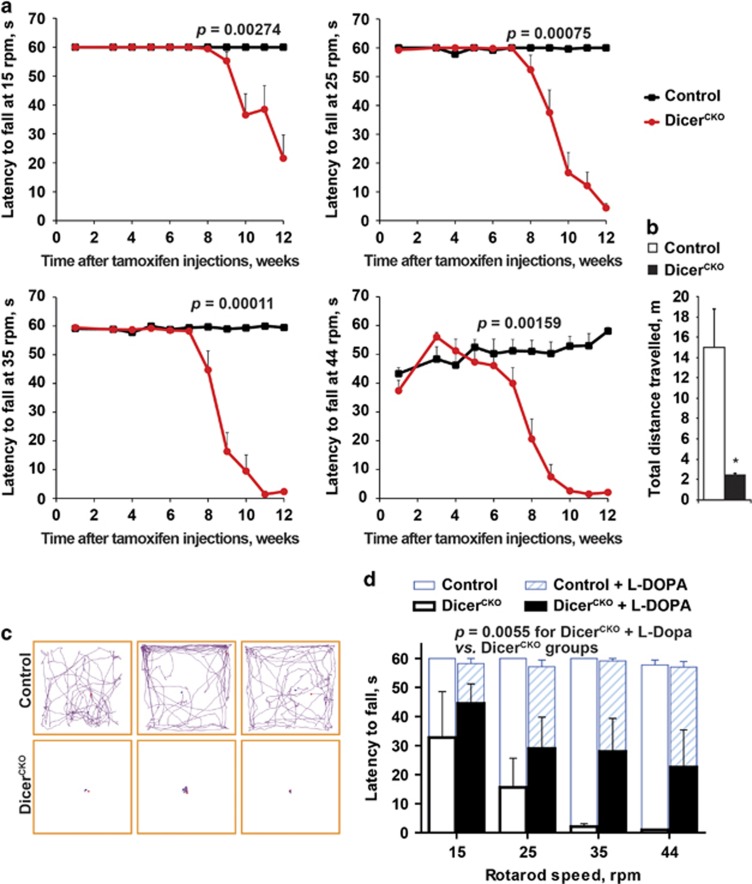
Progressive locomotor abnormalities in Dicer^CKO^ mice. (**a**) Mean latency to fall in a constant speed rotarod assay at 15, 25, 35 and 44 r.p.m. at indicated times after start of Tam injections in control and Dicer^CKO^ mice (*n*=7 and 9, respectively). Automated threshold determination for each speed was followed by a log-rank test to calculate the indicated *P* values. (**b** and **c**) Quantification (**b**) and representative animal tracks (**c**) of the total distance traveled by control or Dicer^CKO^ mice during 5-min open field tests performed 10 weeks after start of Tam injections (*n*=4 and 3, respectively). *P<0.05 as determined by Student’s unpaired two-tailed *t*-test. (**d**) Injection of L-DOPA attenuated locomotor abnormalities in Dicer^CKO^ mice at 17 weeks after start of Tam injections measured by a constant speed rotarod assay at 15, 25, 35 and 44  r.p.m. (*n*=5 and 4 for control and Dicer^CKO^ mice, respectively). The indicated *P*-value was calculated by a two-way repeated measurements ANOVA

As a consequence of the observed loss of the SN DA neurons and striatal DA, Dicer^CKO^ mice exhibited progressive balance and motor coordination abnormalities in a modified constant speed rotarod assay, a highly sensitive method to detect locomotor and balance deficits starting from the early stages of neurodegeneration^29, 36^ (Figure 4a). During the first 6 weeks after start of Tam treatment, all mice tested were able to maintain their balance for 40–60 s at all rotation speeds (Figure 4a). Starting from the 7th week, however, Dicer^CKO^ mice exhibited a progressive decline in performance that was initially detected at the highest rotation speed, 44 rotations per minute (r.p.m.) and also gradually at 35, 25 and 15 r.p.m. during weeks 8 to 10 (Figure 4a). In the 12th week after Tam treatment, Dicer^CKO^ mice failed to maintain balance at higher rotation speeds for >5 s (Figure 4a). The mobility of Dicer^CKO^ mice in the open field test was also greatly reduced at 10 weeks after Tam start (Figures 4b, c and Supplementary Video S1). Notably, these animals exhibited profound involuntary resting tremor, postural and gait impairments and rigidity (Supplementary Videos S1 and S2), consistent with a characteristic complex of PD symptoms.^37^ Similar to PD patients, treatment with L-DOPA significantly improved the locomotor performance of Dicer^CKO^ mice (Figure 4d), confirming that the observed phenotype was due to the lack of DA input within the basal ganglia circuit.

### Stimulation of Dicer activity promotes survival of cultured DA neurons

**Figure 5:**
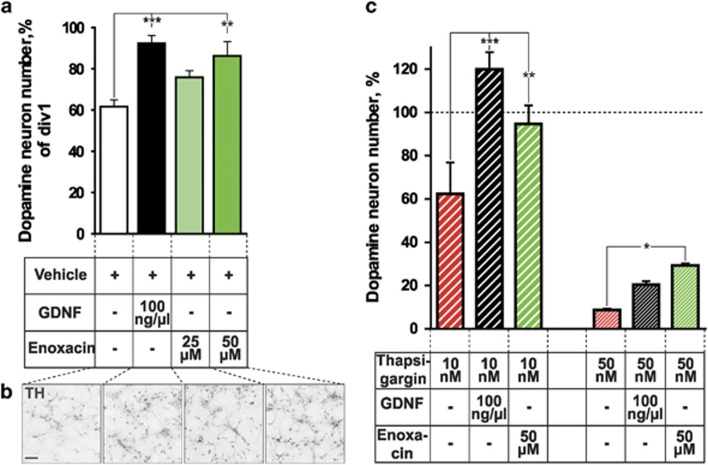
Stimulation of miR biogenesis pathway promotes survival of cultured DA neurons and protects them from thapsigargin-induced ER stress. (**a** and **b**) Counts of TH-positive neurons (**a**) and representative TH immunostaining images (**b**) of primary ventral midbrain cultures from E13.5 mouse embryos after 5 days incubation in cell culture medium supplemented with indicated concentrations of either GDNF, enoxacin or vehicle (*n*=7–10). (**c**) Effect of GDNF and enoxacin on survival of primary ventral midbrain DA neurons treated for 48 h (starting from day 5 *in vitro*) with ER stress inducer thapsigargin (TG) at indicated concentrations. GDNF and enoxacin were added to the cells 6 h prior to TG treatment (*n*=4–6). *P<0.05, **P<0.01 ***P<0.001, as determined by one-way ANOVA followed by Dunnett’s *post hoc* test (**a**) or Holm-Šídák test (**c**)

The observed neurodegenerative phenotype of adult Dicer^CKO^ mice (Figure 3) demonstrates the importance of the miR biogenesis pathway for the survival of DA neurons. We therefore hypothesized that stimulation of miR processing may promote DA neuronal maintenance and evaluated the survival of DA neurons in mouse embryonic midbrain cultures treated with enoxacin – a drug known to stimulate Dicer activity.^19^ GDNF, a potent factor supporting DA neuronal survival,^5^ was used as a positive control. After a 5-day treatment with 50 *μ*M enoxacin, we found an increase in the number of surviving TH-positive DA neurons to similar levels as observed with GDNF treatment (Figures 5a and b). Moreover, similar to GDNF, enoxacin rescued DA neurons after thapsigargin treatment (Figure 5c), but was not effective against 6OHDA and MPP+ (Supplementary Figure S5) at indicated concentrations, suggesting that enoxacin specifically protected DA neurons against ER stress. Thus, although knockout of Dicer was fatal for DA neurons (Figure 3), activation of Dicer and miR biogenesis was neuroprotective (Figure 5).

## Discussion

Our results uncover an age-related downregulation of Dicer in the ventral midbrain. We hypothesized that an age-related and PD-linked decline of Dicer expression may cause deregulation of miR networks and compromise the function of aged DA neurons in a cell-autonomous manner. Indeed, mature miR expression profiles in microdissected DA neurons from young and old mice identified a number of deregulated miRs with about 78% of them downregulated, whereas their precursors remained unchanged in line with our hypothesis of impaired Dicer-dependent miR maturation. Notably, comparison of our miR expression profiles with deep sequencing data on whole brain samples from aged mice^25^ revealed a strong overlap between the two data sets (26 out of 42 downregulated miRs) and strikingly similar expression changes of some of the miRs, suggesting that these may regulate conserved pathways in different neuronal cell populations.

Several miRs, such as miR-133b, miR-7 and miR-153, miR-433, let-7a-5p and miR-184-5p, miR-205, miR-132 and miR-34b/c, have previously been implicated in the development and maintenance of DA neurons and were linked to neurodegeneration.^10, 12, 15, 24^ Although miR-133b appeared to be important in the regulation of Pitx3 during development of DA neurons,^14^ our data do not provide strong support for its role in the maintenance of adult DA neurons, as miR-133b had only a tendency to be downregulated in DA neurons from old mice (*P*=0.055). Expression levels of both let-7a-5p and miR-184-5p in DA neurons from young or old mice were low and remained unchanged. These data suggest that, although these two miRs may promote survival of DA neurons on the background of pathogenic mutations of LRRK2 in transgenic flies,^38^ the normal aging process in mammals does not seem to destabilize their expression. Among the miRs reported to regulate *α*-synuclein, miR-7a-5p did not change and miR-153-3p was upregulated in DA neurons from old mice, however, there was no evidence for a reduction of *Snca* expression (Supplementary Figure S1A).

Our data indicate that the loss of Dicer function affects DA neuronal survival. First, already a heterozygous ablation of Dicer in adult DA neurons resulted in a progressive loss of striatal DA and its metabolites, with apparent differential vulnerability of male *versus* female mice Importantly, *DICER1* appears to also be downregulated in DA neurons from PD patients,^32, 39^ implying that the age-related decline of Dicer function could represent a mechanism contributing to pathogenesis of PD. Similarly, heterozygous mutations of Dicer have been implicated in cancer development^40^ demonstrating that Dicer haploinsufficiency can affect cellular functions. We further show that Dicer is crucially important for neuronal survival, as its homozygous ablation caused degeneration of DA neurons leading to a typical complex of PD-like symptoms, which could be significantly alleviated by L-DOPA application.

Second, stimulation of Dicer activity with enoxacin^19, 26^ promoted survival of cultured embryonic midbrain DA neurons and rescued them from thapsigargin-induced death. In contrast, enoxacin was not effective in protecting DA neurons against 6OHDA and MPP+ at indicated concentrations suggesting its specific activity in counteracting ER stress. This finding is consistent with a protective activity of enoxacin in stress-induced ALS-mutant HEK293 cells and in a mouse model of ALS,^18^ suggesting that a stimulation of miR biogenesis may have general cytoprotective effects on the background of ER stress. Thapsigargin treatment induces ER stress and inhibits Dicer activity by promoting its localization to stress granules.^18^ Interestingly, increased ER stress and deregulation of unfolded protein response (UPR) pathways may also promote age-related decline of DA neurons in PD.^5^ Regulation of ER stress and UPR pathways has been suggested as a possible molecular mechanism of neuroprotection by neurotrophic factors CDNF and MANF promoting survival of DA neurons *in vitro* and *in vivo*,^41, 42^ whereas miRs are also known to regulate neurotrophic factors signaling and ER stress.^43^ In line with the enoxacin-mediated neuroprotection demonstrated in this study (Figures 5a and c), we speculate that, similar to the mechanism proposed for ALS,^23^ the age-related decline of Dicer combined with increased cellular stress in DA neurons may compromise miR biogenesis thus contributing to neurodegeneration in PD. Taken together, our data suggest that disruption of miR biosynthesis in DA neurons through Dicer downregulation or depletion profoundly diminishes DA cell function and survival, whereas its pharmacological stimulation is neuroprotective.

## Materials and methods

### Animal experiments

Mice were maintained on the C57Bl/6 N genetic background with a 12- h light–dark cycle and free access to water and food. The Dicer^fl/fl^/DATCreERT2 (DicerCKO) and Dicer^HET^ mouse lines were generated by mating Dicer^fl/fl^ or Dicer^fl/wt^ mice^30^ with DATCreERT2 mice.^27^ To generate Dicer^fl/fl^/tdTomato/DATCreERT2 mice, Dicer^fl/fl^/DATCreERT2 animals were mated with Cre-reporter Rosa26-tdTomato mice.^34^ Inducible Cre recombinase was activated in 8- to 10-week-old mice by intraperitonial injections of 1 mg Tam (Sigma-Aldrich, St. Louis, MO, USA) diluted in sunflower oil twice daily for 5 consecutive days.^29, 44^ Littermates harboring only floxed alleles were used as controls. All experimental procedures were performed with the approval by the institutional Committee on Ethics of Animal Experimentation (Regierungspräsidium Karlsruhe and national Animal Experiment Board of Finland) and carried out in accordance with the local and European legislation on the protection of animals used for scientific purposes.

### Immunohistochemistry and fluorescent *in situ* hybridization (FISH)

Mice were perfused with 4% paraformaldehyde (PFA); the brains dissected and fixed overnight in 4% PFA and processed for either paraffin or vibratome sections. For cryosections, mice were killed, the brains dissected and immediately frozen on dry ice. Immunohistochemical staining was performed as previously described^28, 29^ using the following antibodies: anti-TH (1:1000, Millipore, Billerica, MA, USA, #AB1542), anti-DOPA decarboxylase (Ddc) (1:1000, Millipore #AB1569), anti-dopamine transporter (Dat) (1:500, Millipore #MAB369) and anti-glial fibrillary acidic protein (GFAP) (1:1000, Millipore #AB5541).

Combined FISH and immunofluorescence staining was performed either on vibratome sections or on cryosections as described in.^45^ Briefly, brain sections were incubated for 30 min at room temperature in 2 × SSC followed by prehybridization for 2 h in hybridization solution containing 50% deionized formamide (Ambion, Foster City, CA, USA), 5 × SSC, 1 × Denhardt’s solution (Applichem, Darmstadt, Germany), 0.25 mg/ml yeast tRNA (Sigma-Aldrich) and 0.5 mg/ml salmon sperm DNA (Sigma-Aldrich), and hybridized overnight at 55° C with 30 nM *Dicer1*-specific 5′- and 3′-DIG-labeled miRCURY LNA detection probe (Exiqon, Vedbaek, Denmark) diluted in hybridization solution. After stringency washes in the solution containing 50% deionized formamide, 1 × SSC, and 0.1% Tween20, sections were washed in 0.2 × SSC and then in 1 × PBS containing 0.1% Tween20, and immunostained with anti-TH antibody (1:500, Cell Signaling, Danvers, MA, USA #2792). The LNA probe was detected with an anti-DIG-POD antibody (1:100, Roche, Indianapolis, IN, USA, #11207733910) and visualized with TSA Plus Cy3 kit (PerkinElmer, Waltham, MA, USA).

### Quantitative RT-PCR

Total RNA isolated from ventral midbrain samples served as a template for DNA synthesis using Super-Script III first-strand synthesis kit (Invitrogen, Carlsbad, CA, USA). For genomic DNA contamination control, samples with no added reverse transcriptase enzyme were included. Quantitative PCR was performed with a CFX96 Real-Time System (Bio-Rad, Hercules, CA, USA) using TaqMan Gene Expression Assays (Life Technologies, Carlsbad, CA, USA) according to the manufacturer’s instructions. The mRNA levels of two housekeeping genes, *Hprt1* and *B2m*, were measured to control for the equal amount of input cDNA.

### Fluorescent labeling and laser microdissection of DA neurons in mice

For retrograde labeling of DA neurons, mice were anesthetized with ketamine/xylazin, placed in a stereotaxic frame (David Kopf Instruments, Tujunga, CA, USA), and received instrastriatal (bregma +0.4 mm; lateral +/–1.8 mm; ventral –3.5 mm) injections of 300 nl red fluorescent retrobeads (Lumafluor, Naples, FL, USA). Animals were killed after 1–2 weeks, the brains dissected and used for cryosectioning. Serial coronal sections (7 *μ*m) of the midbrain spanning the region between bregma –3.4 to –3.6 were mounted on nuclease-free membrane slides (Zeiss, Oberkochen, Germany), fixed in ascending ethanol series and used for laser-assisted microdissection of fluorescently labeled DA neurons in the SN from both sides on the brain as described in.^4^ Individual neurons (approximately 2000 per mouse) were dissected on a Zeiss PALM MicroBeam system (Zeiss) and catapulted into AdhesiveCap tubes (Zeiss). Total RNA including miRs was isolated from the dissected samples with a miRNeasy Micro kit (Qiagen, Hilden, Germany), according to the manufacturer’s instructions.

### MiR expression profiling on DA neurons from young and old mice

High-throughput miR expression profiling was performed using quantitative PCR (qPCR)-based TaqMan miRNA arrays (Applied Biosystems, Foster City CA, USA) according to the manufacturer’s instructions. Briefly, total RNA including miR isolated from the microdissected DA neurons of seven young (10 weeks old) and five old (94 weeks old) mice served as template for cDNA synthesis using Megaplex RT Primer pools A and B. After 12 cycles of pre-amplification PCR with Megaplex PreAmp Primers, 40 cycles of qPCR amplification were performed on TaqMan Array Rodent MicroRNA A and B Cards Set v3.0 using 7900HT Fast Real-Time PCR System (Applied Biosystems).

### Analysis of miR profiling data

Amplification plots were analyzed using SDS2.2 software (Applied Biosystems) with Automatic Baseline and Manual Cq set to 0.2, as recommended by the manufacturer. Cq values were exported to qBase+ software (Biogazelle, Zwijnaarde, Belgium). Global normalization on all detected targets^46, 47, 48^ was performed to calculate relative expression levels for every detected miR, and statistically significant differences between young and old groups were determined by Mann–Whitney test. Histograms and clustering were produced using open source R/Bioconductor packages for the analysis of high-throughput qPCR.^49, 50^

### Quantification of miR precursors

MiR precursor molecules (pri-miR and pre-miR) were quantified using miScript Precursor Assays (Qiagen) according to the manufacturer’s instructions. The same total RNA samples used for mature miR expression profiling served as templates for cDNA synthesis with miScript II RT Kit (Qiagen, Hilden, Germany), using HiFlex buffer for the reaction. For genomic DNA contamination control, samples with no added reverse transcriptase enzyme were included. Quantitative PCR was performed on LightCycler 480 system (Roche, Indianapolis, IN, USA) using QuantiTect SYBR Green PCR Master Mix and miScript Precursor Assay primers targeting stem loops of selected miRs. snRNA RNU6B was used for normalization.

### Behavioral assays and l-DOPA treatment

The modified constant speed rotarod assay was performed as previously described.^29, 36^ After a training period when each mouse attained stable baseline levels of performance staying at 15 r.p.m. for 60 s, mice received several trials at 15, 25, 35 and 44 r.p.m. rotation speed with 60-s maximum trial length and 5-min intervals between individual trials. Two maximal values per speed per day were used to calculate the average, which was used for subsequent statistical analyses.

For the open field test, mice were placed individually into an open arena and monitored for 5 min via video camera. The resulting data were analyzed using the image processing system EthoVision 3.0 (Noldus Information Technology, Wageningen, Netherlands) and Any-maze 4.82 (Stoelting Co., Wood Dale, IL, USA). For each sample, the system recorded position, object area and the status of defined events to calculate the traveled distance.

To follow the effects of L-3,4-dihydroxyphenylalanine (l-DOPA) on locomotor performance, 17 weeks post Tam (wpT) injection control and Dicer^fl/fl^/DATCreERT2 mice were tested in the modified rotarod assay before and immediately after an intraperitoneal injection of 20 mg/kg l-DOPA (Sigma-Aldrich) complemented with 12 mg/kg benserazide (Sigma-Aldrich).

### *In vivo* detection of reactive oxygen species (ROS)

To detect and quantify the production of ROS, 200 *μ*l dihydroethidium (Sigma-Aldrich; 1 mg/ml solution) was administered via the tail vein injection^51^ to 4 weeks post-Tam Dicer^fl/fl^/DATCreERT2 mice and controls as described in Andrews *et al.*^35^ The mice were killed 3.5 h after injection; brains were dissected, fixed overnight in 4% PFA, processed for vibratome sections and immunostained with anti-TH antibody (Millipore). Ethidium fluorescence was visualized using a Zeiss LSM780 confocal microscope.

### Primary embryonic midbrain cultures and enoxacin treatment

Primary cultures of mouse embryonic DA neurons were prepared as previously described;^52^ the cells were plated in 10 *μ*l drop of medium on poly-ornithine coated wells of 96-well plates, and 1 h later 100 *μ*l medium containing either vehicle, GDNF (PeproTech, Rocky Hill, NJ, USA, 100 ng/*μ*l), or enoxacin (Sigma-Aldrich; 25 or 50 *μ*M) were added to each well. Forty-eight hours after plating, half of the medium was replaced with the fresh one containing the same drug concentrations. The cells were fixed 5 days after plating and DA neurons were identified by immunofluorescent staining with anti-TH antibody (1:500; Millipore #MAB318). The plates were imaged with CellInsight CX5 High Content Screening (HCS) Platform (Thermo Fisher Scientific, Inc., Waltham, MA, USA) at × 10 magnification, and the images quantified with CellProfiler software.^53, 54^ Number of counted TH-positive cells was normalized to a number of plated TH-positive cells (counted on the day 1 *in vitro*). The data represent an average of 7–11 wells from two independent experiments.

To assess if enoxacin can rescue oxidative or ER stress-induced cell death, DA cultures were prepared as described above, however, they were not treated with any drugs until DIV5. At DIV5, half of the medium was replaced by the medium containing GDNF (PeproTech, 100 ng/*μ*l) or enoxacin (Sigma; 50 *μ*M); 6 h later thapsigargin (Thermo Fisher), 6OHDA (Sigma) or MPP+ (Sigma) were added to respective wells. Cells were subsequently cultured for 24 h (6OHDA) or 48 h (thapsigargin and MPP+), fixed, stained and counted as described above. Number of TH-positive cells was normalized to control wells at the day of fixation. The data represent an average of 4–6 wells.

### Statistical analysis

Statistical significance was calculated by Student’s two-tailed unpaired *t*-test, Mann–Whitney test, one-way ANOVA followed by Dunnett’s multiple comparison test, two-way ANOVA followed by Bonferroni *post hoc* test or log-rank test using R/Bioconductor packages, qBase+ (Biogazelle) or GraphPad Prism software (GraphPad Software, Inc., La Jolla, CA, USA). Data in text and figures are represented as means ± S.E.M.

For the data from constant speed rotarod assays, we utilized Kaplan–Meier survival analysis after automated threshold determination. As the time the mice could spend on the rod was capped at a maximum of 60 s, the raw time values are of limited information for statistical analysis. We therefore focused on the time period until a relevant decrease in rotarod performance for each mouse could be observed and utilized Kaplan–Meier survival analysis after automated threshold determination. As such, we took the first time point when the time on the rod dropped below a specific threshold value. This threshold value was determined for each rotarod speed in each animal experiment. Healthy trained mice corrected for falls not related to locomotor performance, are able to stay on the rod for 60 s at 15, 25 or 35 r.p.m. and >50% of them fulfill this task also at 44  r.p.m.^29, 36^ Therefore, the threshold value was defined as a mean between the maximum time on the rod (60 s) and the mean time on the rod at the last time point tested (12 weeks after Tam) for all animals in the Dicer^CKO^ group. As such data match survival data in type, log-rank tests were used for analysis and calculation of *P-*values.
