# Supplementary material for: Dicer and microRNAs protect adult dopamine neurons
Source: Cell Death Dis. 2017 May 25;8(5):e2813–. doi: 10.1038/cddis.2017.214 (PMC5520729; doi:10.1038/cddis.2017.214)
Supplement: Supplementary Table and Figures [file cddis2017214x1.pdf]

Dicer and microRNAs protect adult dopamine neurons

Running title: **Dicer protects dopamine neurons**

Piotr Chmielarz[1,2], Julia Konovalova[1], Syeda Sadia Najam[3], Heike Alter[4], Timo Petteri Piepponen[5], Holger Erfle[6], Kai C. Sonntag[7], Günther Schütz[4], Ilya A. Vinnikov[3,4,¶,*], Andrii Domanskyi[1,4,¶,*]

[1] Institute of Biotechnology, P.O. Box 56, University of Helsinki, Helsinki 00014, Finland

[2] Institute of Pharmacology, Polish Academy of Sciences, Department of Brain Biochemistry, 31-343 Krakow, Smetna street 12, Poland

[3] Laboratory of Molecular Neurobiology, Sheng Yushou Center of Cell Biology and Immunology, School of Life Sciences and Biotechnology, Shanghai Jiao Tong University, 800 Dongchuan Road, 200240 Shanghai, China

[4] Molecular Biology of the Cell I Division, German Cancer Research Center, Im Neuenheimer Feld 280, Heidelberg 69120, Germany

[5] Division of Pharmacology and Pharmacotherapy, P.O. Box 56, University of Helsinki, Helsinki 00014, Finland

[6] ViroQuant-CellNetworks RNAi Screening Facility, BioQuant, Im Neuenheimer Feld 267, Heidelberg University, Heidelberg 69120, Germany

[7] Department of Psychiatry, McLean Hospital, Harvard Medical School, 115 Mill Street, Belmont, MA 02478, USA

[¶] I.A.V. and A.D. contributed equally to this work.

[*] Corresponding authors

Ilya A. Vinnikov, ilya.vinnikov@gmail.com, Andrii Domanskyi, andrii.domanskyi@helsinki.fi

SUPPLEMENTAL FIGURES AND TABLES

Table S1. MicroRNAs exhibiting statistically significant changes in microdissected DA neurons from old vs. young mice

						Normalized Cq values											
						Young mice							Old mice				
	p	comparison	ratio	95% ci low	95% ci high	y1	y2	y3	y4	y5	y6	y7	o1	o2	o3	o4	o5
mmu-miR-7a-1-3p	0.017676768	old / young	0.225004479	0.078117461	0.648088335	29.17397	30.75683	28.7133	28.59265	27.88514	28.57092	29.1365	32.39091	30.38667	32.78864	30.92776	29.14397
mmu-miR-342-3p	0.047979798	old / young	0.244265433	0.064654982	0.922830699	26.07443	25.63105	25.90748	25.80598	25.70147	28.22526	25.34639	25.85638	26.04642	29.56816	29.86222	29.32855
mmu-miR-690	0.01010101	old / young	0.269117467	0.102523447	0.706416075	27.6019	28.66463	29.56546	29.46131	28.07038	29.29691	28.65564	32.26029	32.15253	29.42647	29.81319	29.6133
mmu-miR-338-3p	0.01010101	old / young	0.288619226	0.112955697	0.737466631	31.13808	29.90221	31.13236	30.25739	30.05268	32.14366	30.49845	34.40295	31.17499	32.71478	32.99353	31.33814
mmu-miR-148a-3p	0.047979798	old / young	0.294965152	0.099870105	0.871176025	30.43193	29.56285	30.40986	31.11932	29.92483	31.78267	28.87659	32.31794	30.17727	34.24456	32.10199	31.4709
mmu-miR-494-3p	0.017676768	old / young	0.302021609	0.105057464	0.868258651	31.17604	30.77286	29.85826	30.34973	31.77649	31.54064	30.82247	33.29693	35.2331	31.24781	31.56274	31.79329
mmu-miR-204-3p	0.002525253	old / young	0.343624116	0.205411427	0.574834296	30.15714	30.10649	29.42224	29.40018	30.16515	30.03877	28.95118	31.61559	30.28793	31.989	31.61131	30.94533
mmu-miR-652-3p	0.047979798	old / young	0.388090946	0.17563278	0.85755394	26.42761	24.69019	25.38174	27.42606	26.55159	26.81359	26.5842	27.46375	26.52842	27.34597	28.32894	28.49985
mmu-miR-134-5p	0.017676768	old / young	0.39170912	0.206062644	0.744608687	29.75237	31.0145	29.91448	30.15404	30.19808	30.96325	30.25624	30.23131	31.24914	32.58887	31.97229	32.32837
mmu-miR-361-5p	0.047979798	old / young	0.398594114	0.141309472	1.124321428	29.09262	28.55411	29.28782	29.32206	29.06797	28.56732	29.05186	33.32995	30.21306	29.93891	29.33196	28.78099
mmu-miR-668-3p	0.03030303	old / young	0.426255647	0.216656076	0.838628117	29.98941	29.41475	29.31775	29.81828	30.15746	31.0144	29.34313	30.30287	29.89483	31.78602	31.80718	31.68528
mmu-miR-706	0.002525253	old / young	0.430880669	0.272009462	0.682542988	28.14356	28.85748	28.40377	28.47875	28.38489	29.08221	27.45199	29.48134	30.25753	29.25851	29.10738	29.97032
mmu-miR-323-3p	0.047979798	old / young	0.441070156	0.203901878	0.954100495	29.21516	29.1556	30.15013	30.31257	28.97115	29.70526	28.80289	29.36187	29.98697	30.73141	30.78866	32.40194
mmu-miR-770-3p	0.03030303	old / young	0.503783227	0.252354821	1.005717027	30.33429	30.76348	30.31317	29.68172	31.42555	30.36162	30.25081	33.22933	31.24238	30.99534	30.79936	30.91538
mmu-miR-877-3p	0.002525253	old / young	0.518421078	0.365627231	0.735066733	28.20392	28.20661	27.48309	27.89607	27.71517	27.36761	27.89196	28.4852	28.45433	29.39092	29.1422	28.38382
mmu-miR-758-3p	0.03030303	old / young	0.529842376	0.344750971	0.814306462	28.80794	29.67138	29.05812	29.50315	29.21888	29.05306	29.51343	30.20844	29.11825	30.15853	30.7549	30.64596
mmu-miR-532-5p	0.017676768	old / young	0.566872121	0.375671197	0.855386317	27.4694	27.23939	28.01037	27.64003	28.13734	27.94862	27.33014	27.64971	28.1997	29.05549	28.7143	28.88623
mmu-miR-1839-5p	0.03030303	old / young	0.566874602	0.380451542	0.844645845	27.63256	27.95693	28.96489	28.19054	28.17868	28.69285	28.10563	29.59505	29.29303	28.5776	28.67097	29.1879
mmu-miR-219-5p	0.03030303	old / young	0.569165732	0.360696677	0.898122028	28.37211	27.22265	28.14138	27.97104	27.93031	28.3839	27.49682	28.55022	27.93105	29.04674	29.52645	28.66679
mmu-miR-676-3p	0.01010101	old / young	0.586872447	0.387781444	0.888178829	29.339	29.65178	28.69788	29.53123	29.32761	28.29088	29.67501	29.54069	30.22941	29.79173	29.98719	30.37638
mmu-miR-138-1-3p	0.01010101	old / young	0.591421219	0.409081529	0.855035081	29.35226	29.93518	30.07029	29.64764	29.61548	28.92618	29.23086	30.28577	30.75533	30.56998	30.21811	29.65802
mmu-miR-370-3p	0.01010101	old / young	0.592019216	0.4022667	0.871279558	26.31115	27.06438	27.51747	27.38709	26.70345	26.38503	27.05528	28.27873	27.70569	27.44759	27.52284	27.41505
mmu-miR-93-5p	0.03030303	old / young	0.602883387	0.405798022	0.895687901	29.01117	28.21212	28.07025	28.19373	28.4229	28.91114	28.22586	29.35694	28.44854	29.64107	29.57398	28.80626
mmu-miR-467F	0.047979798	old / young	0.60664697	0.351896183	1.045821364	27.66968	27.9131	28.39408	27.4935	27.64428	29.08529	27.03766	29.12954	29.12854	28.33621	28.07013	28.39637
mmu-miR-598-3p	0.03030303	old / young	0.609953729	0.379160372	0.981230052	28.51955	28.72264	29.44719	28.73151	29.01833	28.77334	28.8246	29.22247	28.82837	30.72141	29.20882	29.89734
mmu-miR-543-3p	0.002525253	old / young	0.612381193	0.416722879	0.899904334	27.80893	29.0299	28.99659	28.9054	28.85019	29.12665	28.37004	30.04591	29.31058	29.27865	29.23941	29.29701
mmu-miR-409-3p	0.047979798	old / young	0.627160248	0.425644658	0.924080613	26.74241	27.53088	27.23143	27.35301	26.55752	27.24506	27.16457	27.07722	27.71773	28.35244	27.53656	28.27071
mmu-miR-149-5p	0.002525253	old / young	0.633077336	0.540494367	0.74151913	23.35209	23.78996	23.16046	23.27905	23.28228	23.27347	23.54402	23.96253	23.97509	24.00079	24.1317	24.14286
mmu-miR-146b-5p	0.002525253	old / young	0.639578255	0.503385407	0.812618601	28.4054	28.31253	27.9628	27.70531	27.84766	28.28768	28.41395	28.55161	28.53046	28.85364	28.98547	28.97096
mmu-miR-744-5p	0.005050505	old / young	0.667534594	0.533174865	0.835752891	25.81478	26.16276	25.43796	25.63087	25.45509	25.74131	25.58159	25.95002	26.19266	26.18868	26.45616	26.57388
mmu-miR-212-3p	0.01010101	old / young	0.693138174	0.52875557	0.908624997	26.32527	26.13912	26.34066	25.87276	25.54791	26.48656	25.99629	26.43141	26.58911	26.83645	26.95066	26.34241
mmu-miR-204-5p	0.047979798	old / young	0.703669846	0.514036982	0.963259978	22.64535	22.93977	22.23585	22.68935	22.80631	22.60356	22.32613	23.24161	22.34302	23.27808	23.55117	23.15435
mmu-miR-328-3p	0.03030303	old / young	0.705877569	0.561479564	0.887411002	21.54213	21.92743	21.5022	21.54276	21.87907	21.56239	21.55265	21.87093	21.74459	22.18553	22.41094	22.521
mmu-miR-193b-3p	0.002525253	old / young	0.719741109	0.617206528	0.839309437	25.97525	25.67393	25.51956	25.81675	25.96921	25.6588	25.61746	26.11701	26.03776	26.21079	26.44583	26.29725
mmu-miR-30e-3p	0.005050505	old / young	0.736996898	0.596041982	0.911285521	24.18517	24.34882	24.32482	23.8309	24.13773	24.49614	24.43852	24.48941	24.56311	24.5733	25.10943	24.72474
mmu-miR-191-5p	0.03030303	old / young	0.742533987	0.603780324	0.913174378	23.82249	23.84912	23.62819	23.37534	23.66943	23.42235	23.60055	23.91825	23.60931	24.25408	24.26048	24.22486
mmu-miR-485-3p	0.03030303	old / young	0.742945805	0.601941786	0.916979818	26.85787	26.74272	26.94395	27.13775	26.63608	27.27286	26.55999	27.20506	27.59146	27.21732	27.135	27.38824
mmu-miR-664-3p	0.017676768	old / young	0.755522155	0.627445468	0.909742369	24.77337	25.09619	25.22899	24.86191	24.8826	25.44298	24.97554	25.24466	25.62452	25.39199	25.39605	25.55191
mmu-miR-9-3p	0.01010101	old / young	0.759907945	0.649690783	0.888822961	22.85817	22.80582	22.88428	22.69297	22.76649	23.19192	22.5829	23.27773	23.16461	23.27424	23.38535	23.00898
mmu-miR-487b-3p	0.01010101	old / young	0.776066106	0.649401406	0.927436551	27.40064	27.73153	27.24897	27.73039	27.42226	27.53711	27.6509	27.96199	27.65653	28.20575	27.73262	27.93029
mmu-miR-127-3p	0.01010101	old / young	0.779199467	0.656390031	0.924986335	20.56175	20.99541	20.47519	20.62386	20.8223	20.36399	20.91952	21.08168	20.91553	21.11761	21.05692	21.02939
mmu-miR-125a-5p	0.047979798	old / young	0.806115987	0.631009965	1.029814139	25.27442	25.224	24.95645	25.2266	25.33019	25.24453	25.70316	26.08102	25.51412	25.36002	25.25078	25.74829
mmu-miR-544-3p	0.017676768	old / young	1.373778893	1.106006264	1.706381336	26.77266	27.19487	26.9511	27.06718	26.9469	26.55127	26.8606	26.91935	26.34522	26.19588	26.30959	26.47105
mmu-miR-29b-1-5p	0.01010101	old / young	1.586050471	1.146220727	2.194652425	27.6397	27.83222	27.97253	27.62337	27.16975	27.70336	27.21425	26.24916	27.41576	26.91337	27.15452	26.90799
mmu-miR-380-5p	0.03030303	old / young	1.589548298	1.125282415	2.245359704	27.60103	28.11436	28.6043	28.1679	28.73181	27.60435	28.26411	27.75823	27.79008	27.41266	27.28165	27.19134
mmu-miR-1896	0.047979798	old / young	1.954921467	0.949134133	4.026530929	29.66726	29.00142	29.56488	29.59834	27.0466	29.73501	29.1065	27.26597	28.38082	28.37076	28.43215	28.22904
mmu-miR-130b-3p	0.012121212	old / young	2.12522736	1.217727653	3.70903241	32.44917	33.78066	31.93808	32.41661	32.31787	32.46153	33.05983	NA	31.74968	31.31349	32.10591	31.0083
mmu-miR-153-3p	0.002525253	old / young	2.17164921	1.655144831	2.849333908	32.01108	31.40004	31.91966	31.69699	31.12637	31.70822	31.69236	30.78157	30.04013	30.48913	30.60993	30.73866
mmu-miR-2183	0.017676768	old / young	2.29583983	1.380872655	3.817064888	30.21659	28.84353	30.51236	30.2051	29.68096	29.9738	29.34017	27.67111	28.91302	28.78089	28.88482	28.87828
mmu-miR-1954	0.047979798	old / young	2.475256975	1.025671013	5.973550011	33.46523	31.65555	33.88987	32.35149	30.91429	33.70955	32.77913	30.22402	31.53246	31.60456	31.31739	32.18733
mmu-miR-1961	0.03030303	old / young	3.446144972	1.043968955	11.37573595	36.50852	35.31008	NA	34.93677	34.67674	33.7548	38.24133	33.64534	32.97861	34.76943	33.66825	33.87033
mmu-miR-200b-3p	0.035714286	old / young	4.678017292	1.608194346	13.60772117	34.44555	35.30959	NA	35.22486	33.50568	NA	33.49506	NA	NA	32.68389	32.59701	31.22986
mmu-miR-337-5p	0.03030303	old / young	5.436847303	1.109716181	26.63681858	27.34131	26.80509	26.81994	27.49783	27.3809	24.6565	27.55396	26.75935	27.21179	21.65188	23.92726	22.56123
mmu-miR-25-3p	0.03030303	old / young	7.424529898	1.281373991	43.01916896	29.84025	29.43631	31.32793	31.25647	30.12597	26.46916	30.75662	29.9622	28.97047	24.96992	25.4157	25.65787

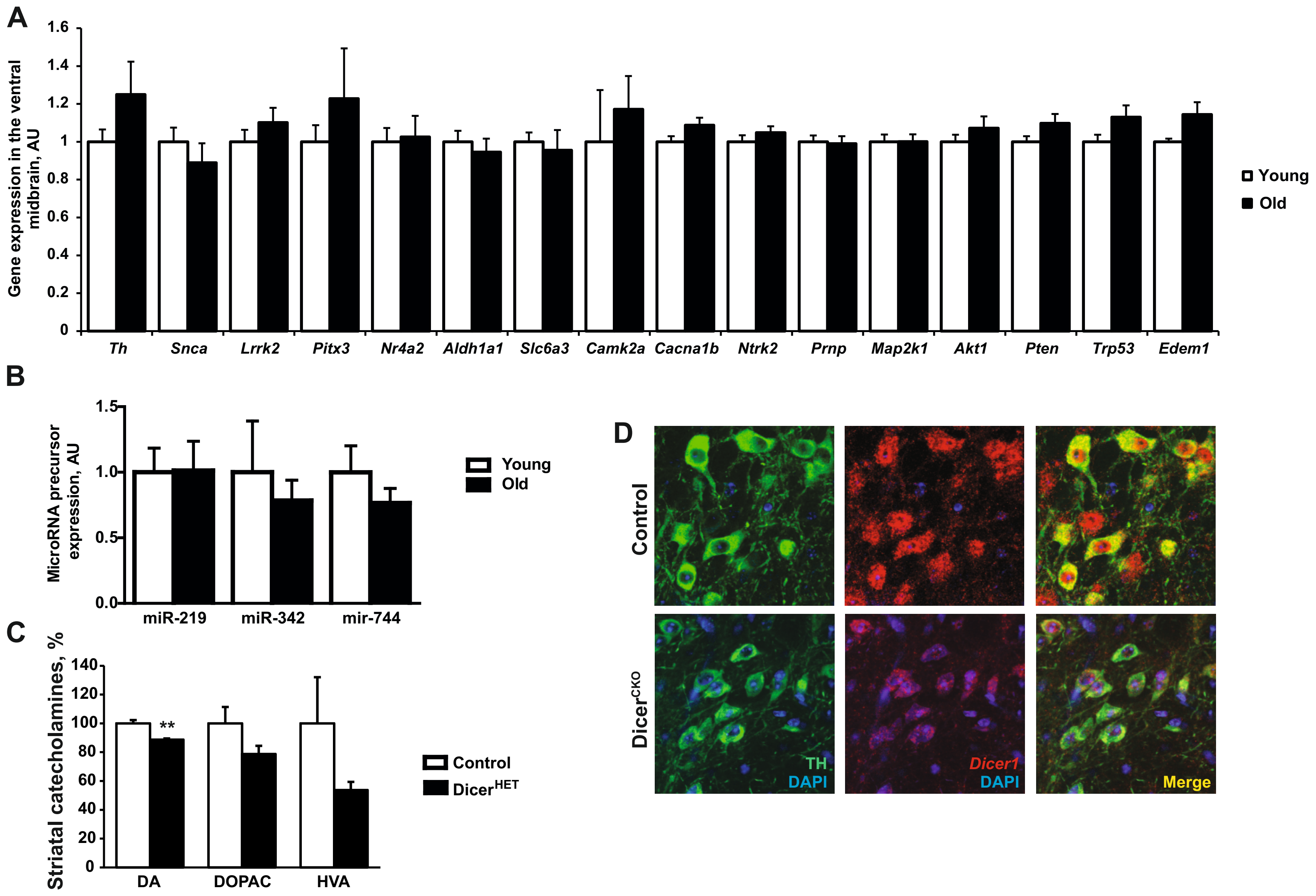

Fig. S1. Relative expression levels of selected RNAs in the ventral midbrain samples from young and old wild-type mice, quantification of striatal catecholamines in Dicer^{HET} females, and loss of *Dicer1* mRNA after Tam injections in Dicer^{CKO} mice
(**A**-**B**) Quantitative PCR analysis of the expression levels of selected mRNAs (**A**) and pri-/pre-miRs (**B**) in the ventral midbrain from young and old wild-type mice. n=6 and 8, respectively. (**C**) Quantification of striatal content of DA and its derivatives DOPAC and HVA in control and Dicer^{HET} female mice (n=5 for both groups) 19 weeks after start of tamoxifen (Tam) injections. (**D**) Loss of *Dicer1* mRNA in DA neurons 2 weeks after start of Tam injections visualized by fluorescent *in situ* hybridization (FISH) with LNA probe (red) and immunofluorescent staining for TH (green). Scale bar, 50 μm.**, $p<0.01$; in comparison to control mice, as determined by Student's unpaired two-tailed *t*-test.

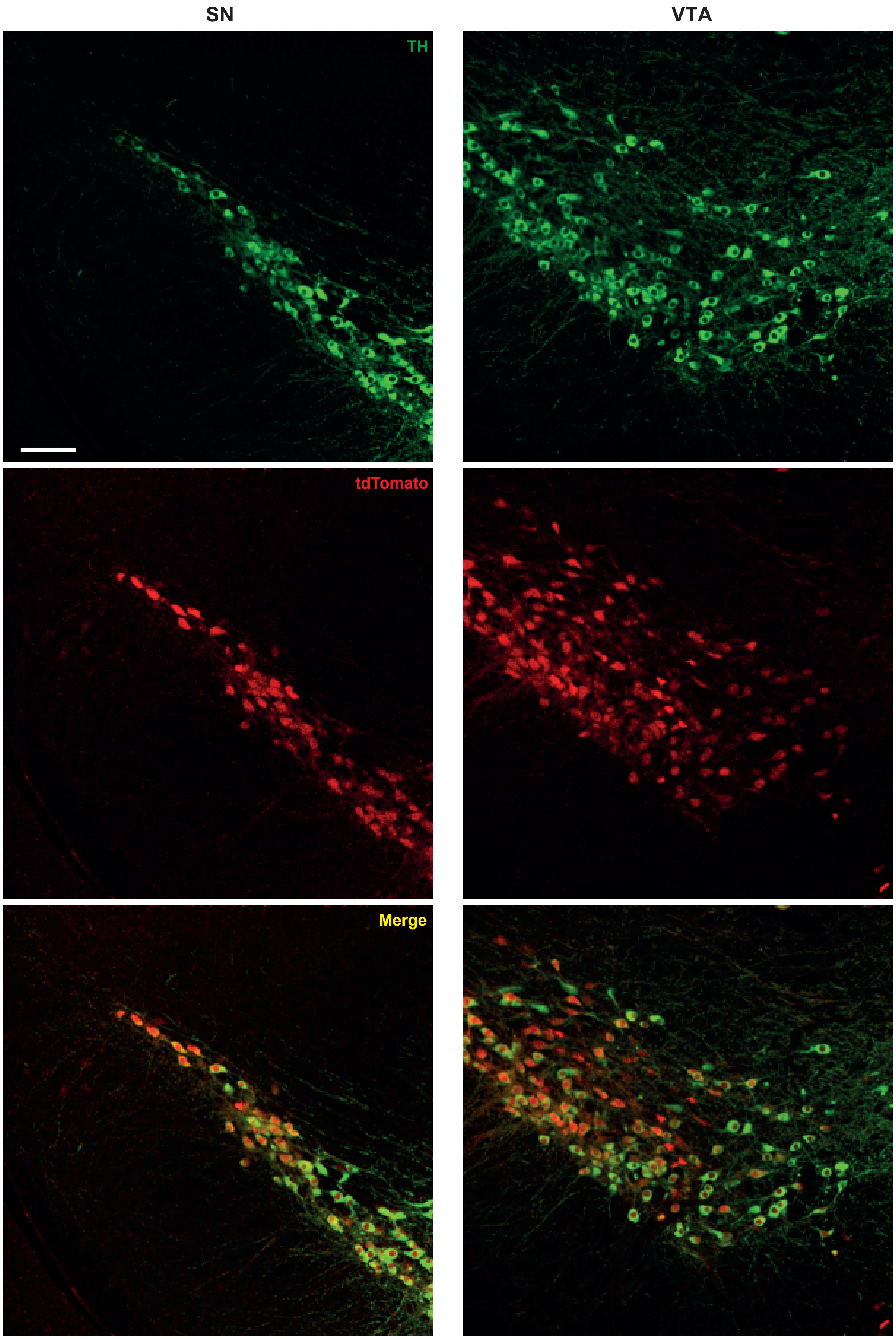

Fig S2. Co-localization of tdTomato and TH after induction of recombination in Dicer$^{fl/wt}$/tdTomato/CreERT2 mice.

Confocal microscopic images of tdTomato fluorescence (red) and TH immunostaining (green) on the ventral midbrain sections from Dicer$^{fl/wt}$/tdTomato/CreERT2 mice 3 weeks after start of Tam injections. Scale bar, 100 μm.

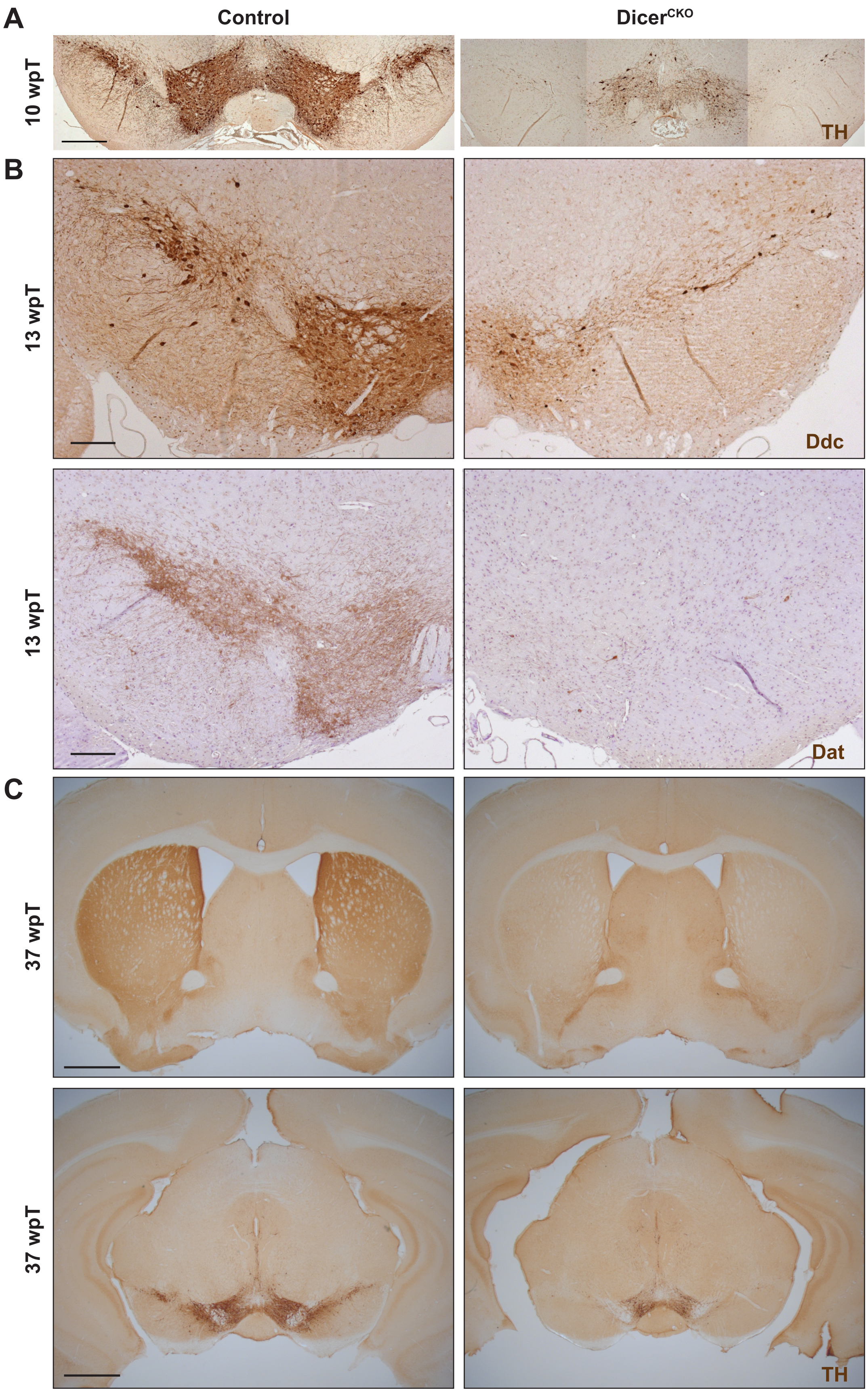

Fig S3. Degeneration of DA neurons in the SN and VTA of Dicer^{CKO} mice 10, 13 and 37 weeks after induction of DA neuron-specific CreERT2-driven ablation of *Dicer1*.

(**A**) Loss of the ventral midbrain DA neurons at indicated times after the induction of *Dicer1* deletion by Tam injections (wpT, weeks post Tam) visualized by TH immunostaining. Scale bar, 500 µm. (**B**) Loss of dopaminergic neurons in ventral midbrain sections from Dicer^{CKO} mice 13 weeks after start of Tam injections visualized by immunostaining for DOPA decarboxylase (Ddc, top) or dopamine transporter (Dat, bottom). Scale bar, 200 µm. (**C**) Loss of DA neurons and their striatal projections in Dicer^{CKO} mice 37 weeks after start of Tam injections visualized by TH immunostaining. Scale bar, 1000 µm.

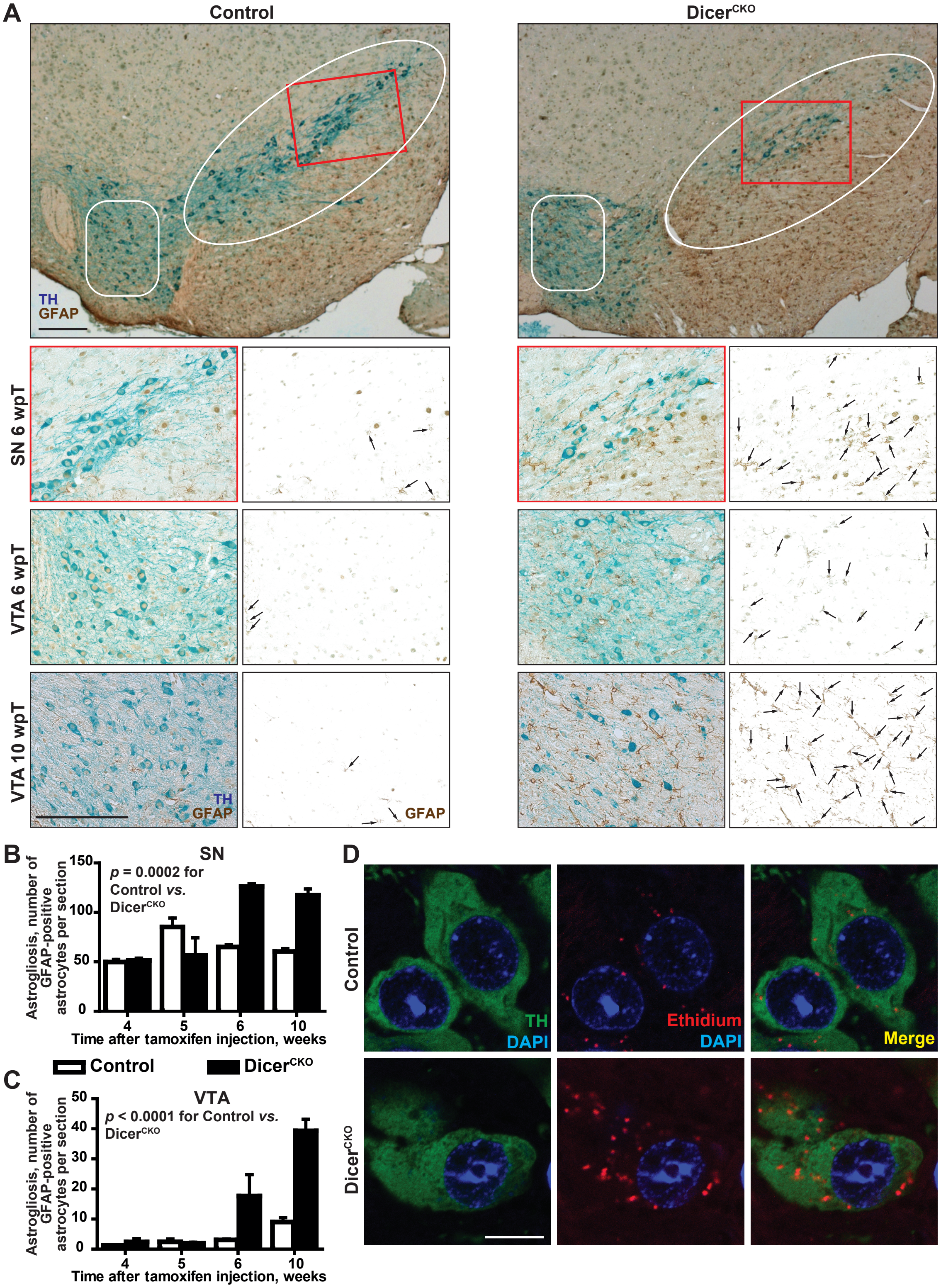

Fig S4. Astrogliosis and increased oxidative stress in Dicer^{CKO} mice.
(**A**) Astrogliosis in the substantia nigra (SN) and the ventral tegmental area (VTA) of Dicer^{CKO} mice 6 and 10 weeks after start of Tam injections visualized by immunostaining for glial fibrillary acidic protein (Gfap; brown). DA neurons are visualized by TH immunostaining (blue). Areas of the SN and VTA used for astroglyosis quantification are outlined by white ovals or rounded rectangles, respectively. Scale bar, 500 µm. (**B, C**) Counts of Gfap-positive cells in the SN and VTA in control and Dicer^{CKO} mice at indicated time points after start of Tam injections. n = 3, 4, 4, 4 for control and 2, 2, 2, 3 for Dicer^{CKO} mice, respectively, for the indicated time points. Statistical significance was calculated by an unpaired 2-way ANOVA test. (**D**) The mice received an injection of 200 µl dihydroethidium to the tail vein 4 weeks after start of Tam treatment. Increased oxidative stress in DA neurons of Dicer^{CKO} mice was visualized by an increase in ethidium fluorescence (red) in TH-immunostained DA neurons (green). Scale bar, 10 µm.

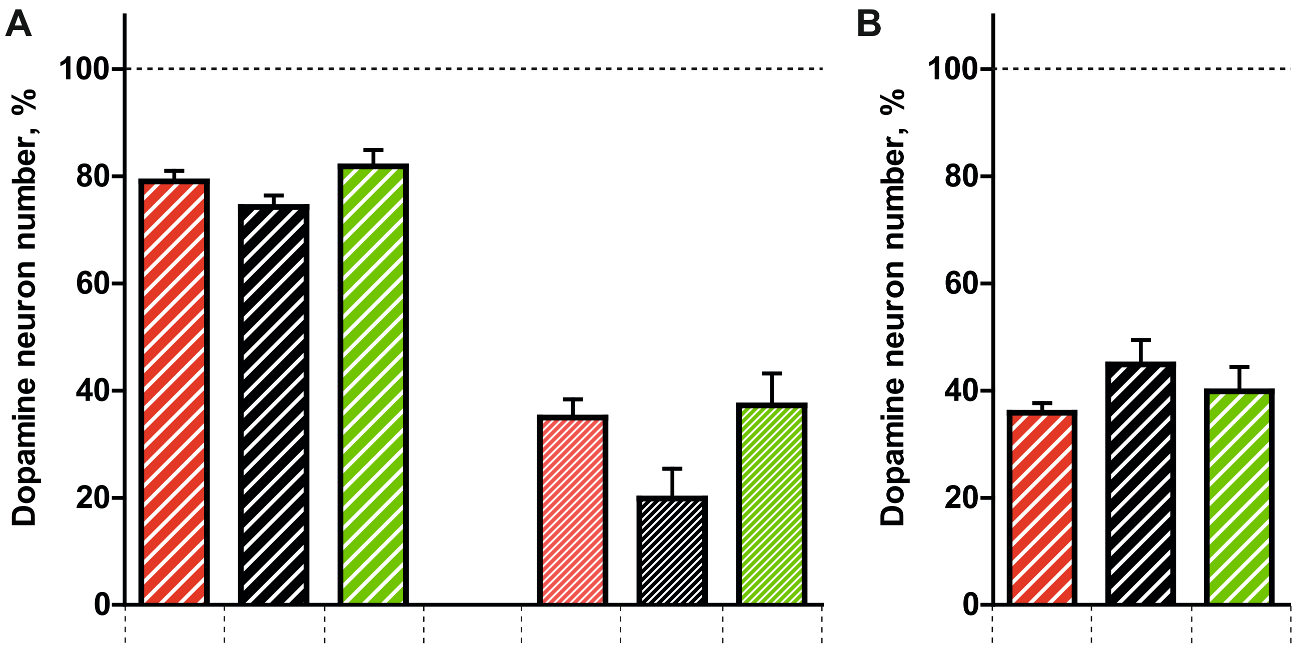

6OHDA	15 µM	15 µM	15 µM		30 µM	30 µM	30 µM
GDNF	-	100 ng/µl	-		-	100 ng/µl	-
Enoxacin	-	-	50 µM		-	-	50 µM

MPP+	1.5 µM	1.5 µM	1.5 µM
GDNF	-	100 ng/µl	-
Enoxacin	-	-	50 µM

Fig S5. Enoxacin is not effective in protecting cultured DA neurons from 6OHDA or MPP+. (**A, B**) Effect of GDNF and enoxacin on survival of primary ventral midbrain DA neurons treated (starting from day 5 *in vitro*) for 24 hr with 6OHDA (**A**) or for 48 hr with MPP+ (**B**) at indicated concentrations. GDNF and enoxacin were added to the cells 6 hr prior to the stressor treatment (n=4-6).
